# Integrated peloton and fruiting body isotope data shed light on mycoheterotrophic interactions in *Gastrodia pubilabiata* (Orchidaceae)

**DOI:** 10.1007/s00572-025-01213-8

**Published:** 2025-06-11

**Authors:** Kenji Suetsugu, Hidehito Okada

**Affiliations:** 1 Department of Biology, Graduate School of Science, Kobe University, Kobe, Hyogo 657-8501 Japan; 2 Institute for Advanced Research, Kobe University, 1-1 Rokkodai, Nada-Ku, Kobe, Hyogo 657-8501 Japan

**Keywords:** Metabarcoding, Mycoheterotrophy, Mycorrhiza, Nutritional mode, Orchidaceae, Peloton, Stable isotopes

## Abstract

The mycoheterotrophic nutritional mode, characterized by the acquisition of fungal-derived carbon by plants, has long captivated botanists and mycologists. Recent stable isotope analyses of fungal pelotons isolated from roots have advanced our understanding of this nutritional strategy; however, concerns remain regarding potential isotopic biases, particularly ^15^N depletion during lysis or extraction. To address these concerns, we focused on *Gastrodia pubilabiata*, a fully mycoheterotrophic orchid that associates with saprotrophic fungi. This species offers an ideal system to test whether peloton tissues reliably reflect fungal isotope signatures, as its mycorrhizal roots occasionally occur in direct contact with the fruiting bodies of its fungal partner. We measured δ^13^C and δ^15^N values in the aboveground tissues of *G. pubilabiata*, pelotons extracted from its roots, and fruiting bodies of the associated wood-decaying fungus *Cyanotrama gypsea*, along with neighboring autotrophic reference plants. The stable isotope analysis revealed that δ^13^C values were nearly identical between pelotons and fruiting bodies, while δ^15^N values were slightly higher in pelotons, indicating that peloton-derived isotopic data reliably reflect the fungal source. Moreover, the ^13^C and ^15^N enrichment observed in the orchid relative to the fungal fractions was broadly consistent with expected trophic-level fractionation, suggesting a predator–prey-like mode of nutrient transfer. Taken together, these findings support the validity of recently developed isotope-based approaches using extracted pelotons to represent fungal isotopic signatures, at least within this system.

**Supplementary Information:**

The online version contains supplementary material available at 10.1007/s00572-025-01213-8.

## Introduction

All orchids rely on mycorrhizal fungi for carbon during germination and early seedling development, a condition referred to as initial mycoheterotrophy (Merckx 2013; Jacquemyn & Merckx 2019). Some orchids transition to full autotrophy upon reaching maturity; others maintain partial mycoheterotrophy throughout adulthood; still others remain fully mycoheterotrophic for their entire lifespan (Gebauer & Meyer 2003; Gebauer et al. 2016).

The mycoheterotrophic nutritional mode, including partial forms, can be evaluated using stable isotope analysis, particularly of ^13^C and ^15^N (reviewed by Hynson et al. 2013), as fungal tissues are typically enriched in these isotopes relative to photosynthetic plants (Gebauer & Dietrich 1993; Gleixner et al. 1993). Mycoheterotrophic plants often exhibit ^13^C and ^15^N enrichment equal to or exceeding their fungal associates (Schiebold et al. 2017), reflecting nutrient uptake via hyphal degradation or direct transfer (Bougoure et al. 2014; Kuga et al. 2014).

Gebauer and Meyer (2003) proposed a linear two-source mixing model to estimate the contributions of fungus-derived carbon and nitrogen to partially mycoheterotrophic orchids, using autotrophic plants as a 0% reference and fully mycoheterotrophic species as 100%. However, subsequent studies have shown that ^13^C and ^15^N enrichment levels in fully mycoheterotrophic plants vary with the isotopic profiles of their mycobionts and, to a lesser extent, with physiological differences in nutrient assimilation (Hynson et al. 2016; Schiebold et al. 2017). Such variability may lead to under- or over-estimation of fungal carbon contributions in partially mycoheterotrophic orchids (Gebauer et al. 2016; Schweiger et al. 2018).

Although some researchers use the isotopic composition of fungal fruiting bodies associated with partially mycoheterotrophic orchids to define ^13^C values representing the fully mycoheterotrophic endpoint (Yagame et al. 2012; Schiebold et al. 2017), this approach is often limited when the fungi do not produce detectable fruiting bodies or when fruiting bodies are not spatially or phenologically aligned with the orchids (Gomes et al. 2023; Zahn et al. 2023). As a result, studies frequently use fruiting bodies of different species from the same ecological guild (e.g., saprotrophic or ectomycorrhizal fungi) as proxies (Yagame et al. 2012), a practice that may compromise accuracy due to both inter- and intra-specific variation in fungal isotopic signatures.

To overcome these limitations, recent investigations have measured stable isotope ratios in fungal pelotons (intracellular hyphae residing within orchid roots) extracted directly from root tissues (Gomes et al. 2023; Zahn et al. 2023; Suetsugu et al. 2025). While this method circumvents the need to locate fruiting bodies, concerns persist as to whether peloton samples accurately reflect the isotopic composition of the entire fungal organism, particularly concerning ^15^N. In some species, pelotons exhibit δ^15^N values substantially lower than those of orchid tissues. For example, a 12.6 ± 1.7‰ difference has been reported between pelotons and leaves in *Epipactis leptochila*, exceeding the expected isotopic enrichment per trophic level (approximately + 0.8–1.0‰ for δ^13^C and + 2.2–3.4‰ for δ^15^N; Gomes et al. 2023; Zahn et al. 2023). While the large discrepancy in ^15^N enrichment between pelotons and orchids may result from the selective transport of ^15^N-enriched compounds from fungi to orchids, an alternative explanation is the selective retention of ^15^N-depleted components, such as chitin, in pelotons during fungal lysis or peloton extraction (Zahn et al. 2023). If extracted hyphae lose their original isotopic composition, accurately assessing fungal isotope signatures remains difficult, even with labor-intensive peloton isolation.

In this study, we focused on *Gastrodia pubilabiata*, a fully mycoheterotrophic orchid that associates with saprotrophic fungi (Kinoshita et al. 2016). This species provides an ideal system to test whether peloton tissues reliably reflect fungal isotope signatures, as its mycorrhizal roots are among the few known to occur in direct contact with the fruiting bodies of their fungal partner. This rare and spatially explicit association offers a unique opportunity to compare the isotopic composition of pelotons and fruiting bodies derived from the same fungal individual under natural conditions. By targeting *G. pubilabiata* individuals attached to fruiting bodies of *Cyanotrama gypsea* on decaying conifer logs, we compared δ^13^C and δ^15^N values between pelotons extracted from roots and *C. gypsea* fruiting bodies to assess whether they exhibit similar isotope signatures. We also evaluated whether *G. pubilabiata* shows the expected isotopic enrichment relative to *C. gypsea* fruiting bodies and pelotons, as predicted per trophic level. Finally, high-throughput DNA sequencing was performed to confirm the predominant mycorrhizal partner status of *C. gypsea* in the *G. pubilabiata* specimens analyzed.

## Materials and methods

### Study site and sampling scheme

**Fig. 1:**
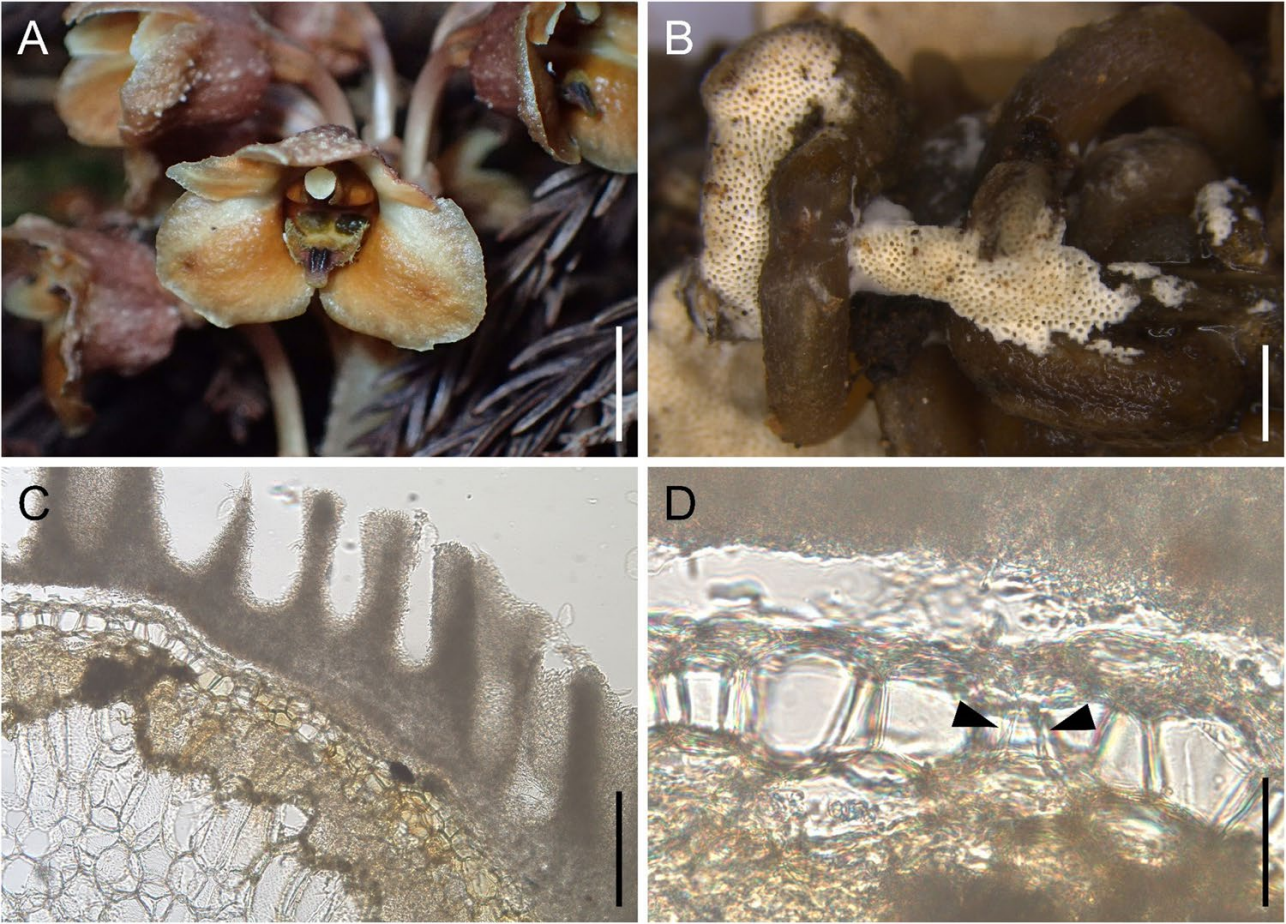
Interaction between *Gastrodia pubilabiata* and *Cyanotrama gypsea.* (**A**) A flowering plant of *G. pubilabiata*. (**B**) Roots of *G. pubilabiata* with an attached *C. gypsea* fruiting body. (**C**) Cross-section of a *G. pubilabiata* root with an attached *C. gypsea* fruiting body. (**D**) The interface between the *G. pubilabiata* root and *C. gypsea* fruiting body. Arrows indicate *C. gypsea* mycelium penetrating the middle cortical cells through the exodermal and outer cortical layers of *G. pubilabiata*. Scale bars: 5 mm (**A**), 2 mm (**B**), 500 μm (**C**), and 100 μm (**D**)

Fieldwork was conducted on November 23, 2023, in a population of *G. pubilabiata* located in Obuchi, Fuji City, Shizuoka Prefecture, Japan. The study site was a warm-temperate forest dominated by *Cryptomeria japonica*. The population consisted of approximately 30 *G. pubilabiata* individuals. Similar to other *Gastrodia* species, *G. pubilabiata* possesses thick, starch-filled rhizomes that lack fungal colonization (Martos et al. 2009; Kinoshita et al. 2016). In contrast, its slender roots, often embedded within decaying wood, are predominantly colonized by its associated fungi (Kinoshita et al. 2016). Within the population, *G. pubilabiata* mycorrhizal roots were frequently observed adhering to the fruiting bodies of *C. gypsea* on decayed *C. japonica* logs (Fig. 1).

Four 1 m × 1 m quadrats were established around a *G. pubilabiata* individual. From these quadrats, we collected aboveground parts and mycorrhizal root samples from each *G. pubilabiata* plant for stable isotopic analysis, peloton extraction, and molecular identification of mycorrhizal fungi. Fruiting bodies of *C. gypsea* and leaf samples from four co-occurring plant species were also collected from each quadrat for stable isotopic analysis, following the criteria of Gebauer and Meyer (2003).

Pelotons were extracted from each *G. pubilabiata* mycorrhizal sample for isotopic analysis, following the methods of Gomes et al. (2023) and Zahn et al. (2023) with slight modification. Briefly, longitudinal sections of mycorrhizal roots were made using a razor blade, then sonicated in deionized water for 15 min to release pelotons. The suspension was transferred to a separate tube using a pipette, and this process was repeated with fresh water until the solution became clear. Pelotons were purified using stacked stainless-steel sieves (250, 106, and 53 µm; Sanpo, Tokyo, Japan). Sieve surfaces were thoroughly rinsed with deionized water to minimize contaminants, and most pelotons were retained by the 106-µm sieve. Retained pelotons were pipetted into Eppendorf tubes, centrifuged for 10 s, and the supernatant was discarded.

### Molecular identification of mycorrhizal fungi

Root fragments (3–5 mm) containing pelotons were taken from each *G. pubilabiata* individual (n = 4) and surface-sterilized. DNA was extracted using a CTAB method (Doyle & Doyle 1990). Representative sections were examined with an Olympus BX-51 microscope (Olympus, Tokyo, Japan) to confirm intracellular colonization.

Following Suetsugu & Okada (2025), we amplified the ITS region of fungal DNA using the ITS86 F/ITS4 primer set fused with 3–6-mer nucleotides and Illumina forward/reverse sequencing primers in the first PCR, followed by the addition of Illumina P5/P7 adapters and sample-specific indices in the second PCR. The pooled library was sequenced on an Illumina MiSeq platform using the MiSeq Reagent Micro Kit v2 (300 cycles). Raw sequence data were deposited in the NCBI Sequence Read Archive (accession no. PRJNA1235933).

Bioinformatic processing was performed using Claident v0.9.2024.06.10 (Tanabe & Toju 2013), following Suetsugu & Okada (2025). In brief, high-quality reads were clustered into operational taxonomic units (OTUs) at a 97% similarity threshold. Taxonomic assignments were made to the genus level where possible using Claident, and OTUs corresponding to known orchid mycorrhizal fungi (Dearnaley et al. 2012; Wang et al. 2021) were retained for further analysis. A maximum-likelihood phylogeny of the dominant OTU and its closest relatives was reconstructed using IQ-TREE v2.2.2 (Minh et al. 2020), with model selection via ModelFinder (Kalyaanamoorthy et al. 2017). Branch support was evaluated using SH-aLRT and ultrafast bootstrap (UFboot).

### δ^13^C and δ^15^N analysis

In total, 28 specimens were sampled, including 4 *G. pubilabiata* aboveground parts, 4 *G. pubilabiata* pelotons, 4 *C. gypsea* fruiting bodies, and 16 autotrophic reference plants (Table S1). These specimens were desiccated at 60 °C for 2 days and finely ground using an agate mortar. The natural abundances of ^13^C and ^15^N were measured at the Research Institute for Humanity and Nature (Kyoto, Japan) using a Delta XP mass spectrometer (Thermo Fisher Scientific, Waltham, MA, USA) coupled to a Flash EA 2000 elemental analyzer (Thermo Fisher Scientific). The relative abundances of the stable isotopes were calculated as:

$$\delta^{13}\mathrm C\;\mathrm{or}\;\mathrm\delta^{15}\mathrm N=\left(R_{\mathrm{sample}}/R_{\mathrm{standard}}\mathit-1\right)\times1,000\left[\permille\right],$$

where *R*_sample_ represents the ^13^C/^12^C or ^15^N/^14^N ratio in the sample, and *R*_standard_ represents the corresponding ratio for Vienna PeeDee Belemnite or atmospheric N_2_, respectively. The C and N isotope ratios were calibrated using the following laboratory standards: CERKU-01 (DL-alanine, δ^13^C = −25.36‰, δ^15^N = −2.89‰), CERKU-02 (L-alanine, δ^13^C = −19.04‰, δ^15^N = 22.71‰) and CERKU-03 (glycine, δ^13^C = −34.92‰, δ^15^N = 2.18‰), which are traceable back to the international standards (Tayasu et al. 2011). Analytical standard deviations were less than 0.09‰ for ^13^C (n = 24) and less than 0.22‰ for ^15^N (n = 24). Total C and N concentrations were determined based on sample weights and corresponding gas concentrations (CO_2_ and N_2_) derived from laboratory standards (Tayasu et al. 2011). Differences in δ^13^C and δ^15^N values among *G. pubilabiata* aboveground parts, *G. pubilabiata* pelotons, *C. gypsea* fruiting bodies, and autotrophic reference plants were analyzed using linear models. Pairwise comparisons were conducted using post hoc Tukey–Kramer tests. All statistical analyses were performed using R software (R Core Team 2025).

## Results

### Morphological and molecular identification of mycobionts

Cross-sections of *G. pubilabiata* roots bearing attached *C. gypsea* fruiting bodies revealed that mycelium extending from the fruiting body penetrates the middle cortical cells through the exodermal and outer cortical layers of the orchid root (Fig. 1). This demonstrates that the root and fruiting body are not merely in contact; rather, *G. pubilabiata* roots can directly establish mycorrhizal associations with fungal fruiting bodies on deadwood, enabling nutrient acquisition from decomposing substrates. Fungal colonization was primarily restricted to the middle cortical layers, with the epidermis and outer cortex remaining largely uncolonized.

Community profiling using metabarcoding confirmed that *G. pubilabiata* predominantly forms symbiotic relationships with *C. gypsea* in the investigated population. After quality filtering, two OTUs were detected in the mycorrhizal tissues of *G. pubilabiata*, comprising 379,404 sequencing reads. The *Cyanotrama* OTU was overwhelmingly dominant, accounting for 99.98% of all reads (379,342 reads), while the other OTU, assigned to Ceratobasidiaceae, represented only 0.02% (62 reads). The *Cyanotrama* sequence showed 100% identity with registered sequences of *C. gypsea* (IDs: KT203291.1, LC631671.1, LC631685.1), the latter two listed as *Neoantrodiella gypsea*, a synonym of *C. gypsea*. Phylogenetic analysis confirmed that the dominant OTU formed a strongly supported monophyletic clade with *C. gypsea* and a previously reported mycobiont of *G. pubilabiata* (Fig. S1).

### Stable isotope analysis

The aboveground tissues of *G. pubilabiata*, *G. pubilabiata* pelotons, and *C. gypsea* exhibited significantly higher δ^13^C values (−21.0 ± 0.2‰, −21.7 ± 0.1‰, and −21.6 ± 0.3‰, mean ± SD, respectively) than those of autotrophic reference plants (−34.9 ± 1.5‰; *P* < 0.001 for all comparisons; Table S2). In contrast, no significant differences in δ^13^C values were observed among *G. pubilabiata* aboveground tissues, pelotons, and *C. gypsea* (*P* > 0.8 for all comparisons).

**Fig. 2:**
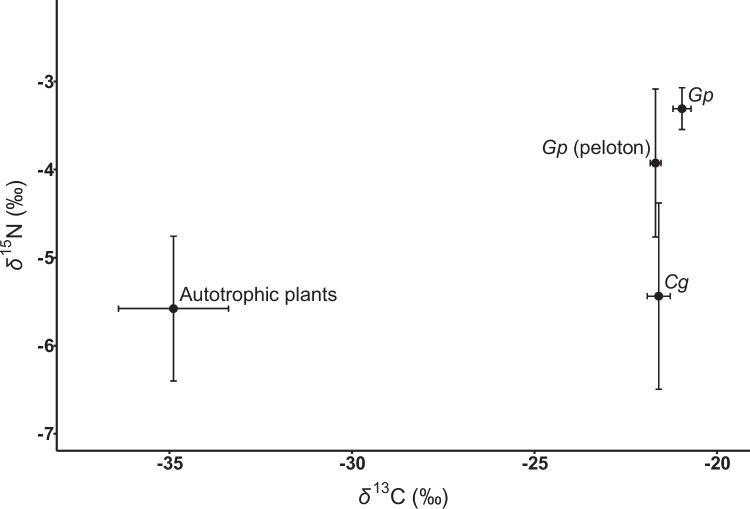
Mean (± SD) δ^13^C and δ^15^N values of *Gastrodia pubilabiata* aboveground tissues, *G. pubilabiata* pelotons, its host fungus *Cyanotrama gypsea,* and neighboring autotrophic reference plants. *Gp*: *Gastrodia pubilabiata*; *Cg*: *Cyanotrama gypsea*

Similarly, *G. pubilabiata* aboveground tissues and pelotons (−3.3 ± 0.2‰ and −3.9 ± 0.8‰, respectively) exhibited significantly higher δ^15^N values than autotrophic reference plants (−5.6 ± 0.8‰; *P* < 0.001 and *P* < 0.005, respectively). In contrast, *C. gypsea* (−5.4 ± 1.1‰) did not differ significantly from autotrophic reference plants in δ^15^N values (*P* = 0.99; Fig. 2). No significant difference was observed between δ^15^N values of *G. pubilabiata* aboveground tissues and pelotons (*P* = 0.70). However, both *G. pubilabiata* aboveground tissues and pelotons exhibited significantly and marginally significantly higher δ^15^N values than *C. gypsea* (*P* < 0.01 and *P* = 0.06, respectively).

## Discussion

Here we show that the pelotons and fruiting bodies of the fungal partner share similar ^13^C and ^15^N isotopic signatures in *G. pubilabiata*, which forms an unusually close association with the fruiting body of its mycorrhizal fungus *C. gypsea* (Hymenochaetaceae). This finding validates the reliability of peloton-based stable isotope analysis, a method that, despite its growing use, has lacked direct empirical support from systems where pelotons and fruiting bodies of the same fungus can be compared under natural conditions (Gomes et al. 2023; Zahn et al. 2023; Suetsugu et al. 2025).

Metabarcoding-based community profiling confirmed that *G. pubilabiata* at our study site primarily associates with *C. gypsea*, consistent with direct observations of roots in contact with its fruiting bodies. While *Gastrodia* species often interact with fungal rhizomorphs in decomposing litter (Lee et al. 2015), this study shows that *G. pubilabiata* can also form mycorrhizal associations directly with fruiting bodies on deadwood, suggesting an alternative nutrient acquisition strategy.

Previous studies have documented diverse fungal associations across *Gastrodia* species (e.g., association with litter-decaying *Mycena* in *G. confusa* or wood-decaying *Armillaria* in *G. elata*) (Ogura-Tsujita et al. 2009; Suetsugu et al. 2020). Although *G. pubilabiata* typically associates with litter-decaying fungi (e.g., Mycenaceae and Marasmiaceae) (Kinoshita et al. 2016), its ability to also form associations with wood-decomposing fungi such as *Cyanotrama* may facilitate its colonization across diverse habitats, including bamboo thickets, cedar plantations, and *Castanopsis* forests.

Stable isotope analyses further confirmed reliance on a wood-decaying fungus in the *G. pubilabiata* samples examined. These samples showed marked ^13^C enrichment relative to co-occurring autotrophic plants (13.9 ± 0.3‰). The values exceed those reported for mycoheterotrophic orchids associated with ectomycorrhizal fungi (7.8 ± 1.6‰, n = 163) or litter-decaying fungi (8.2 ± 0.5‰, n = 15), but align with those dependent on wood-decaying fungi (11.0 ± 2.3‰, n = 15) (Zahn et al. 2022) and with its fungal host *C. gypsea* (13.3 ± 0.4‰). Similarly, *G. flavilabella*, which inhabits coniferous forests and associates with wood-decaying *Hydropus*, exhibits high ^13^C (12.1‰) and ^15^N (4.7‰) enrichment (Lee et al. 2015). These patterns likely reflect the higher δ^13^C values of wood-decaying fungi compared to those of litter-decaying or ectomycorrhizal fungi due to the more ^13^C-enriched substrates of wood (Hobbie et al. 1999; Högberg et al. 1999; Kohzu et al. 1999).

Importantly, δ^13^C and δ^15^N values in pelotons extracted from *G. pubilabiata* closely matched those of *C. gypsea* fruiting bodies. Previous studies have expressed concern that peloton extractions may underestimate δ^15^N due to the preferential retention of cell wall components, such as chitin, which are ^15^N-depleted relative to fungal proteins (Gomes et al. 2023; Zahn et al. 2023). However, our results reveal no indication of such depletion; δ^15^N values were even higher in pelotons than in fruiting bodies. Due to the non-significant difference and limited sample size (n = 4), we acknowledge that it remains unclear whether the observed difference is meaningful at this stage. Anyway, the ^13^C and ^15^N enrichment detected in *G. pubilabiata* tissues relative to pelotons (0.7 ± 0.4‰ for δ^13^C and 0.6 ± 1.0‰ for δ^15^N) and *C. gypsea* fruiting bodies (0.6 ± 0.5‰ for δ^13^C and 2.1 ± 1.2‰ for δ^15^N) roughly aligns with typical trophic-level enrichment patterns observed in consumers relative to their dietary sources (Minagawa & Wada 1984; McCutchan et al. 2003). These patterns suggest that *G. pubilabiata* primarily derives carbon and nitrogen from its mycorrhizal associate in a manner analogous to typical predator–prey nutrient transfer.

In conclusion, our results from a system where both pelotons and fruiting bodies are accessible support the broader use of peloton-based isotope analysis in mycoheterotrophic studies, particularly when fruiting bodies are unavailable. Given the close δ^13^C and δ^15^N values between pelotons and fruiting bodies in *G. pubilabiata*, the pronounced ^15^N enrichment reported in some mycoheterotrophic plants relative to their pelotons (e.g., 12.6 ± 1.7‰ in *Epipactis leptochila*) (Gomes et al. 2023) is more likely attributable to physiological or biochemical specialization, such as selective assimilation of ^15^N-enriched compounds, rather than a methodological artifact.

The question of why some mycoheterotrophic plants exhibit minimal ^15^N enrichment while others show substantial enrichment remains unresolved, as (i) our study focused on a single orchid–fungus association, and (ii) the system examined showed minimal ^15^N enrichment relative to the fungus. Broader isotopic studies across multiple mycoheterotrophic species, incorporating analyses of pelotons and, where feasible, fungal fruiting bodies, are needed to clarify how fungal identity and orchid physiology influence ^15^N enrichment. Although beyond the scope of this field-based study, future experiments using labeled substrates and peloton extraction would further elucidate carbon and nitrogen exchange in orchid–fungus symbioses.

## Supplementary Information

Below is the link to the electronic supplementary material.

Supplementary file1 (XLSX 14 KB)

Supplementary file2 (PDF 118 KB)

## Data Availability

The sequence data are deposited in the NCBI Sequence Read Archive (accession no. PRJNA1235933). Additional information is provided in the Supporting Information section.
