# Supplementary material for: Integrated peloton and fruiting body isotope data shed light on mycoheterotrophic interactions in Gastrodia pubilabiata (Orchidaceae)
Source: Mycorrhiza. 2025 Jun 11;35(3):43. doi: 10.1007/s00572-025-01213-8 (PMC12158851; doi:10.1007/s00572-025-01213-8)
Supplement: Supplementary file 2 — Supplementary file2 (PDF 118 KB) [file 572_2025_1213_MOESM2_ESM.pdf]

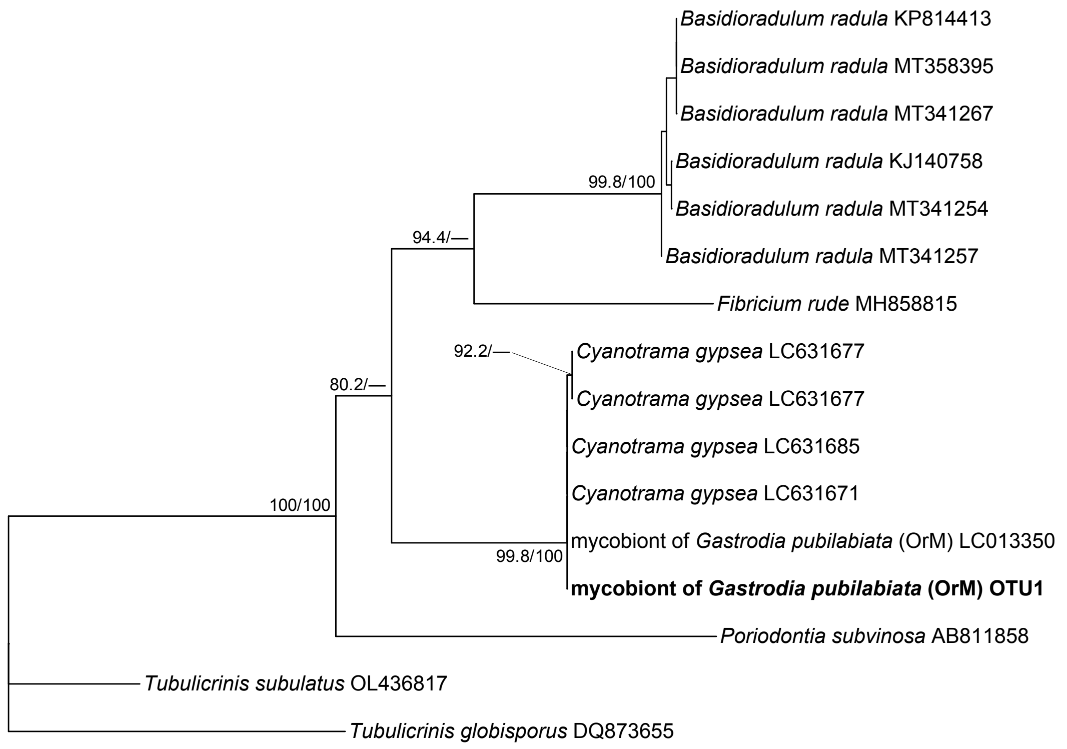

Fig. S1. Phylogenetic tree of ITS2 rDNA sequences from the predominant OTU detected in mycorrhizal samples of *Gastrodia pubilabiata* (in bold), along with sequences retrieved from the INSDC database. The tree is rooted with *Tubulicrinis subulatus* and *Tubulicrinis globisporus* (Tubulicrinaceae). Nodes with SH-aLRT values < 80% and ultrafast bootstrap values < 95% are not shown. The scale bar represents the number of substitutions per site. OrM: Orchid mycorrhizal fungi.
